# Thermodynamic and Computational (DFT) Study of Non-Covalent Interaction Mechanisms of Charge Transfer Complex of Linagliptin with 2,3-Dichloro-5,6-dicyano-1,4-benzoquinone (DDQ) and Chloranilic acid (CHA)

**DOI:** 10.3390/molecules27196320

**Published:** 2022-09-25

**Authors:** Ahmed H. Bakheit, Rashad Al-Salahi, Abdulrahman A. Al-Majed

**Affiliations:** Department of Pharmaceutical Chemistry, College of Pharmacy, King Saud University, P.O. Box 2457, Riyadh 11451, Saudi Arabia

**Keywords:** Linagliptin (LNG), DDQ, Chloranilic acid (CHA), Charge transfer complex (CT), (QTAIM), (NCI), (RDG), UV–Vis spectrophotometer

## Abstract

This study describes the non-covalent interactions of the charge transfer complex (CT), which was responsible for the synthesis of Linagliptin (LNG) with 2,3-Dichloro-5,6-Dicyano-1,4-benzoquinone (DDQ), or with Chloranilic acid (CHA) complexes in acetonitrile (MeCN) at temperatures of (25 ± 2 °C). Then, a UV–Vis spectrophotometer was utilized to identify Linagliptin (LNG) from these complexes. For the quantitative measurement of Linagliptin in bulk form, UV–Vis techniques have been developed and validated in accordance with ICH criteria for several aspects, including selectivity, linearity, accuracy, precision, LOD, LOQ, and robustness. The optimization of the complex synthesis was based on solvent polarization; the ratio of molecules in complexes; the association constant; and Gibbs energy (ΔG°). The experimental work is supported by the computational investigation of the complexes utilizing density functional theory as well as (QTAIM); (NCI) index; and (RDG). According to the optimized conditions, Beer’s law was observed between 2.5–100 and 5–100 µM with correlation coefficients of 1.9997 and 1.9998 for LGN-DDQ and LGN-CHA complexes, respectively. For LGN-DDQ and LGN-CHA complexes, the LOD and LOQ were (1.0844 and 1.4406 μM) and (3.2861 and 4.3655 μM), respectively. The approach was successfully used to measure LGN in its bulk form with high precision and accuracy.

## 1. Introduction

**Figure 1:**
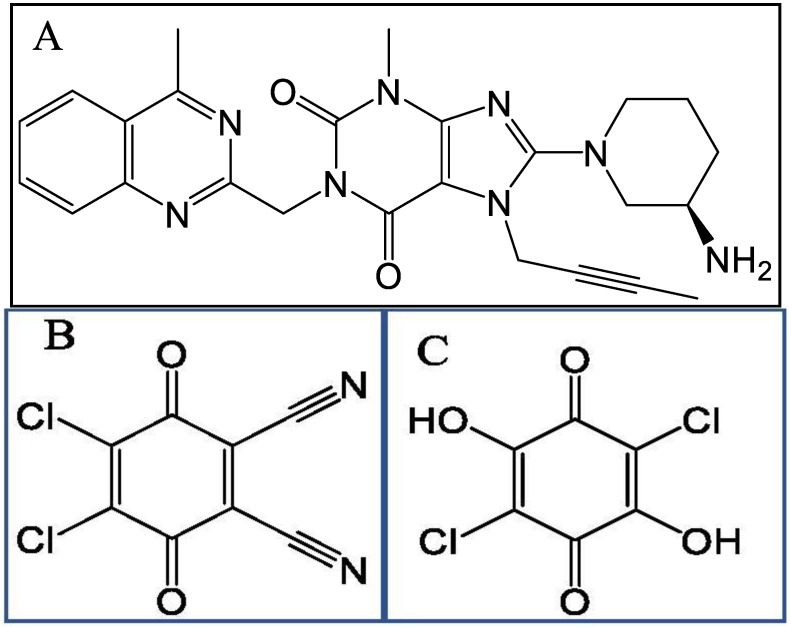
Chemical structure of (**A**) Linagliptin (LNG), (**B**) 2,3-dichloro-5,6-dicyano-*p*-benzoquinone (DDQ) and (**C**) Chloranilic acid (CHA).

Linagliptin (LNG), 8-[(3R)-3-aminopiperidin-1-yl]-7-(but-2-yn-1-yl)-3-methyl-1-[(4methylquinazolin-2-yl)methyl]-3,7-dihydro-1H-purine-2,6-dione], is a selective, competitive dipeptidyl peptidase-4 (DPP-4) inhibitor, that was approved in 2011 by USA, Japan and Europe for the treatment of type 2 diabetes (Figure 1A) [1]. It can be used as a monotherapy or in combination with other common antidiabetic medications including metformin, sulfonylurea, pioglitazone or insulin. It is commercially available as 5 mg film-coated tablets and as 2.5 mg in combination with 850 mg metformin-film-coated tablets. There are many approaches to explain how acceptors and electron-donors molecules interact to produce intensity-colored charge transfer (CT) complex, which absorb in visible light. The term “charge transfer phenomena” was introduced by Mulliken and Foster [2,3,4]. There are numerous *p*-benzoquinone derivatives that are extensively utilized as π acceptors in the production of different CT complexes involved in energy storage that play a crucial role in biological activity. Colored reagents are essential for the quantitative investigation of several medicinal compounds that can act as electron donors [5]. In general, charge transfer includes the transfer of electrons from the donor molecule to the benzoquinone molecule as acceptors, resulting in the formation of a benzoquinone radical anion, which then forms a free radical ion pair or ion pair [5,6]. 2,3-dichloro-5,6-dicyano-p-benzoquinone (DDQ) (Figure 1B) and Chloranilic acid (CHA) (Figure 1C) are well-known benzoquinone derivatives that act as powerful π acceptors in the complexation of anion radicals. Many donor bases are produced in stable free-ion pair complexes as a result of their work [5,6,7]. The binding of that free radical ion or ion pair is dependent on chemical interactions, which are mostly non-covalent [8]. Similarly, non-covalent interactions (NCI) play an essential role in molecule stabilization, as evidenced by the isotropy in crystal packing. According to previous studies, NCI such as halogen bonds, hydrogen bonds, CH⋯π, π⋯π, and weak van der Waals contacts are diffusely distributed in crystal structures [9,10,11,12,13,14,15,16]. Yang et al. [8] introduced the reduced density gradient (RDG) function based on electron density ρ(r), which provides a simple, straightforward, and practical way for researching NCI. The colored 3D gradient isosurfaces and 2D graphs of the decreased gradient versus sign(λ_2_)ρ provide a scientific tool for discussing NCI in molecules. Hirshfeld surfaces and 2D fingerprint plots, which are another powerful tool for examining NCI, are also commonly used in crystal research [17,18]. Using the electron distribution may clearly highlight intermolecular interactions. The non-covalent interactions of complex formation are correlated with the development of intensity-coloured complexes that absorb visible-spectrum radiation.

A comprehensive review of the available literature found that a number of analytical techniques, such as spectrophotometric [19,20,21,22,23,24,25,26,27,28,29,30], colorimetric [31,32,33], spectrofluorometric [33,34,35], and high-performance liquid chromatographic [36,37,38,39] methods, were described for the detection of linagliptin. It has been determined that colorimetric is more specific than spectrophotometry (UV region). The methods used to analyse the LGN singly or in combination are cited in references [19,20,21,22,23,24,25,26,27,28,29,30], which were based on the molecular absorption technique. These methods are sensitive, but they are not as selective as the method being studied. It is one of the justifications for conducting this experiment. In addition, the references [31,32,33] use colorimetric techniques. For example, Moradi et al. [31] carried out an experiment for the ultra-trace quantitative colorimetric determination of anti-diabetic drugs based on gold nanoparticles. The cost of the experiment’s materials is high. Finally, the chromatographic technique was cited in references [36,37,38,39]. This technique is expensive and time-consuming. The reason for this study was that it was suitable for both bulk samples and pharmaceutical samples in terms of how selective it was, how long it took, how easy it was to use, and how much it cost.

The main objectives of this research are to analyze the characteristics of non-covalent intermolecular interactions, particularly the H-bond association between LNG with DDQ and CHA during the synthesis of the CT complex using density functional theory (DFT). In order to comprehend the nature of the non-covalent intermolecular interactions between LNG with DDQ or CHA, TD-DFT, NCI index, IE, RDG, MESP, or QTAIM analyses were performed. Additionally, the effect of solvents and stoichiometry on the bonding association strength is investigated. Moreover, the CT complexes’ association constants and electronic spectra were measured.

In this study, we investigated the charge transfer process as well as the ion pair interaction of LGN with DDQ, or with CHA in (MeCN) and used it to determine LGN spectrophotometrically. In order to identify the bond type, thermodynamic equations are used to calculate the physical characteristics. Specifically, a DFT analysis enables us to assess the geometrical characteristics of the optimised structures of the reactants and complexes. Fukui function analysis, Mulliken atomic charge distribution, topological analysis, electron densities on frontier molecular orbitals, and reactivity parameters all provided information that was equivalent to experimental data.

## 2. Results and Discussion

### 2.1. Optimization of the Reaction Conditions

**Figure 2:**
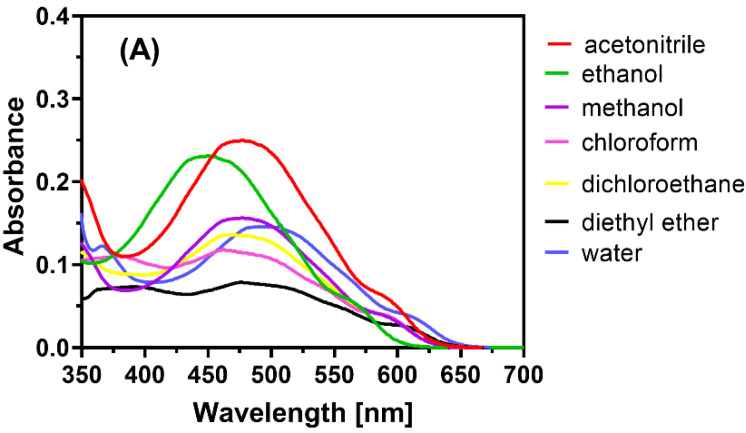

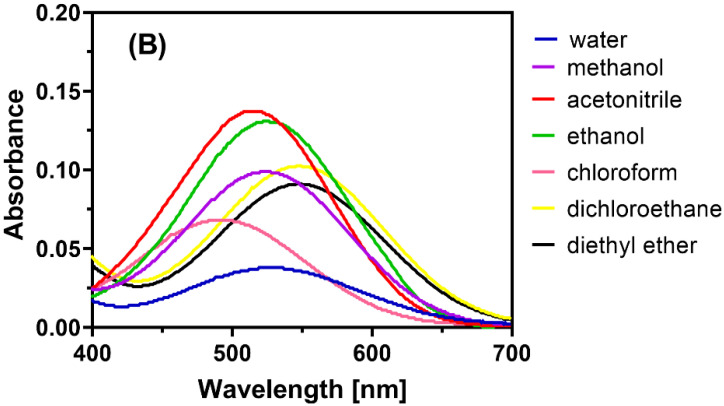
Effect of solvent on the absorption spectrum of the complexes of LGN 50 with (**A**) DDQ and (**B**) CHA.

The charge transfer reaction of LGN with DDQ, or with CHA, was conducted in multiple solvents with varying dielectric constants [40] and polarity indexes to find the best solvent for reaction and colour development. The absorption spectrum of the generated colour complexes was measured and recorded for this purpose (Figure 2). 

**Figure 3:**
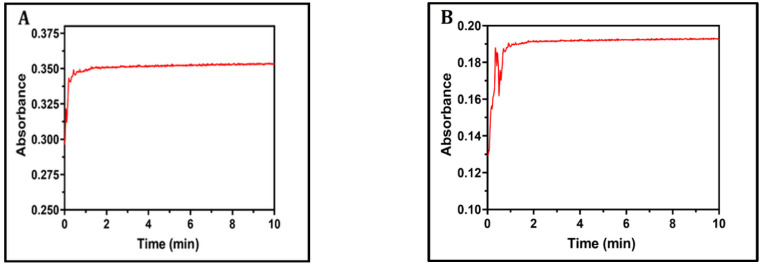
The absorbance of the reaction mixture of LGN (1 × 10^–4^ M) with DDQ (1 × 10^–4^ M), measured at 487 nm (**A**), and with CHA (1 × 10^–4^ M) measured at 514 nm (**B**), and plotted against the corresponding reaction time.

There were slight shifts in the λ_max_ values and variations in the absorbance values. When the reaction was carried out in ethanol (EtOH) and MeCN, which have high polarity and high dielectric constants, the results were better than when the reaction was performed in a solvent with low polarity and low dielectric constants (e.g., diethyl ether and dichloromethane). This phenomenon may be a result of the favourable charge transfer from donor LGN to acceptors DDQ, or with CHA to produce the free radical ions, as well as the interactions of those free radical ions through the transfer of the non-paired electron of oxygen atoms on DDQ and CHA molecules (hydrogen bond donors) to polar hydrogen in LGN molecules (hydrogen bond acceptors) in polar liquids such as MeCN, where the best solvent was found. In all of the next steps, MeCN was chosen as the solvent. The reaction in MeCN occurred immediately at room temperature (25 ± 2 °C), as determined by analysing the influence of time on the process (Figure 3).

### 2.2. Observation of CT Electronic Spectra

**Figure 4:**
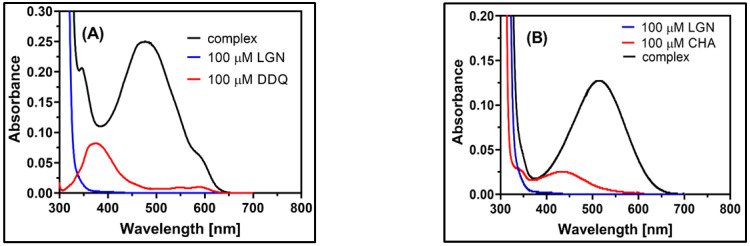
Electronic absorption spectra of (**A**) blue line for LGN (1 × 10^–4^ M), red line for DDQ 1 × 10^–4^ M and complex with black line of LGN-DDQ (1:1); (**B**) blue line for LGN (1 × 10^–4^ M), red line for CHA 1 × 10^–4^ M and complex with black line of LGN-CHA (1:1) complex (1 × 10^–4^ M + 1 × 10^–4^ M) in MeCN at room temperature.

As seen in Figure 4, the spectrum of complexes is completely distinct from those of the individual donor and acceptor spectra, validating the nature of complexes. The spectra of the LGN-DDQ and LGN-CHA complexes contained multi-charged transfer bands in the polar solvents MeCN at 487.50 nm and 514.00 nm, respectively, and the wavelength of the highest absorbance peak was determined for both the complexes. According to the results of a time study conducted on the formation of the complexes, the absorbance has not changed over the course of the experiment, as shown in Figure 3. Instantaneous development of a consistent reddish-brown colour is mostly attributed to the formation of a hydrogen bond between oxygen atoms in the carbonyl groups in the DDQ and CHA, with a hydrogen atom in the amine group in the LGN (Schemes S1 and S2).

### 2.3. Physical Composition of the CT Complex

**Figure 5:**
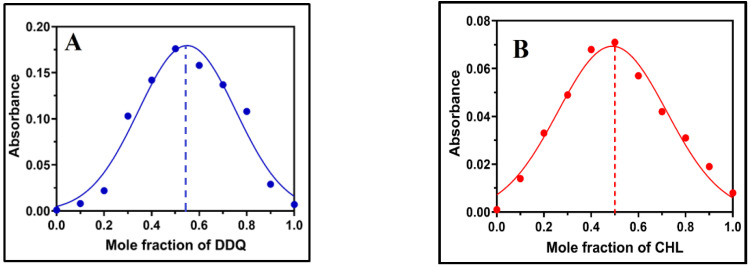
Job’s plot for complexes formation of LGN with (**A**) DDQ (**B**) CHA in MeCN (λabs for DDQ complex (487.52 nm) and CHA complex (514 nm, C sum = 1 × 10^–4^ M).

**Figure 6:**
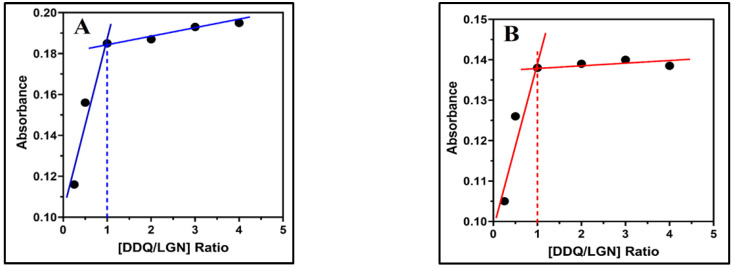
Spectrophotometric plot of LGN (6 × 10^–5^ M) complexes: (**A**) LGN-DDQ and (**B**) LGN-CHA complexes in MeCN (λ_abs_ for DDQ (3 × 10^–5^ M)) complex (487.52 nm) and CHA (3 × 10^–5^ M) complexes.

The molecular composition of the complexes of LGN-DDQ and LGN-CHA were determined by applying Job’s continuous variations at 487.52 and 514 nm [41] to both complexes in (MeCN) medium shown in (Figure 5A,B, respectively). Where maximum absorbance achieved at 0.5 mol fraction indicating 1:1 LGN/DDQ and LGN/CHA stoichiometry for the complexes. The (Figure 6A,B) represents photometric titration plots in MeCN solvents. Here, the results from both complexes indicate the molar ratio of LGN/DDQ and LGN/CHA complexes are 1:1.

### 2.4. Association Constant, Free Energy Change and Ionization Potential

**Figure 7:**
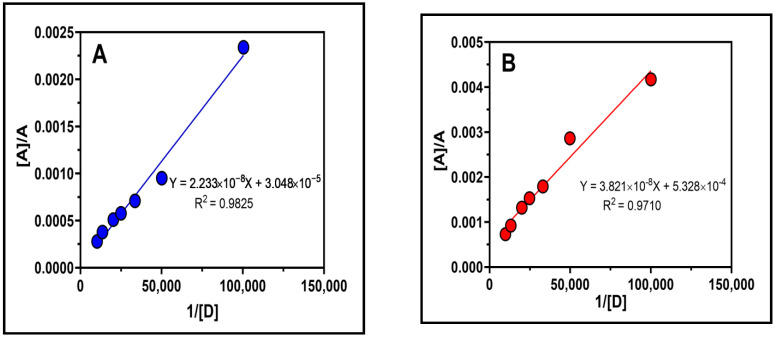
LGN complexes with (**A**) DDQ and (**B**) CHA are shown in this Benesi–Hildebrand plot. On the graph are presented the linear regression equation and the correlation coefficient (r). [A], A, and [D] denote, respectively, the molar concentrations of LGN, the absorbance’s of the complex reaction mixture, and the molar concentration of DDQ and CHA.

The Benesi–Hildebrand approach [42] was used to calculate the association constant (K_c_) at room temperature (25 ± 2 °C) and at the λ_max_ of the formed LGN-DDQ and LGN-CHA complexes using the absorption spectra of the complexes formed by reacting numerous concentrations of DDQ and CHA with a fixed concentration of LGN. A straight line was attained from which the LGN-DDQ and LGN-CHA complex association constant were calculated (Figure 7A,B). The Benesi–Hildebrand method for the determination of K_C_ for donor-acceptor association and ε_C_ values is given below:

(1) $$\frac{\left[D\right]}{A}=\frac{1}{K_{c}\varepsilon_{c}}\times \frac{1}{\left[A\right]}+\frac{1}{\varepsilon_{c}},$$

where [A] and [D] are the initial concentrations of the acceptor and donor, respectively, A is the absorbance of the charge transfer complexes at K_C_ against the solvent as reference, and ε_C_ the extinction coefficient of the complexes. The values of the association constant of LGN-DDQ and LGN-CHA complexes were revealed to be 1.47 × 10^12^ L mol^−1^ and 4.91 × 10^10^ L mol^−1^, respectively.

The complex’s standard free energy change (ΔG^0^) is proportional to its logarithm of formation constant and may be calculated using the formula:

(2) $$\Delta G^{0}=-2.303\times \mathrm{RT}\times {\mathrm{logK}}_{c},$$

where ΔG^0^ is the standard free energy change of the complex (Kilo Calories; Kcal mol^−1^), R is the gas constant (8.314 KJ mole^−1^), T is the absolute temperature in Kelvin (°C + 273) and Kc is the complex association constant (L mol^−1^). ΔG^0^ values of the LGN-DDQ and LGN-CHA complexes were −16.593 Kcal mol^−1^ and −14.5798 Kcal mol^−1^, respectively. This ΔG^0^ value indicates that the interaction between LGN with DDQ and CHA was straightforward, and the complexes were fairly stable [43].

### 2.5. Computational Analysis 

#### 2.5.1. Interaction Energies (IE)

The DFT approach at the B3LYP level with the 6-311G(d,p) basis set has been widely used to obtain the binding energies of the two complexes. In this research, the interaction of LGN with DDQ or CHA were investigated as a charge transfer complex. In the gas phase and in molar ratio (1:1). The complexation energy (∆E) raw complexation energy (∆E) corrected, and the basis set superposition error (∆BSSE) of the two complexes were calculated and the result is shown in Table 1. LGN–DDQ and LGN–CHA complexes have the lowest (∆E)_raw_, ∆EBSSE, and (∆E)_corrected_, indicating that both complexes’ stable.

#### 2.5.2. Non-Covalent Interaction (NCI) Index

**Figure 8:**
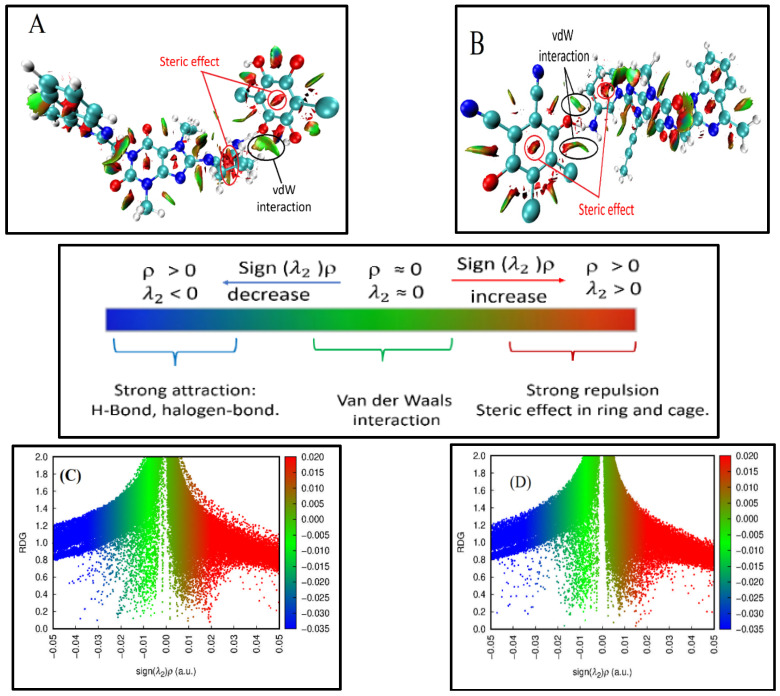
Non-covalent interactions analysed through the RGD method. (**A**,**B**) The RDG isosurface computed for LGN-CHA and LGN-DDQ, respectively, the LGN cation and the CHA and DDQ anion; (**C**,**D**) the RDG scatter plot color-graded for LGN-CHA and LGN-DDQ, respectively, in accordance with the interaction type: strong attraction (blue), weak interaction (green), and strong repulsion (red).

The non-covalent interaction analysis of the LGN^+•^ with DDQ^–•^, or with CHA^–•^ free radical ions, which were produced from the charge transfer reaction of the LGN donor molecule to the DDQ and CHA acceptor molecules (Schemes S1 and S2), has been performed with the reduced density gradient (RDG) method (see Figure 8). Green areas of the RDG isosurface indicate the presence of weak interactions between the LGN^+•^ and 1,4-benzoquinone rings in the DDQ^−^^•^ and CHA^−^^•^, as well as weak interactions between the lone pair electron in the O7 atom of 1,4-benzoquinone and the σ-hole in the hydrogen atoms H48 of LGN. Red areas in the rings indicate strong repulsion effects. The bright blue area between an LGN molecule’s hydrogen H49 and the O71 atom of a carbonyl group in CHA indicates attraction between these two atoms (the H49-O71 distance is 1.915 Å). The same attraction exists between H49 a-hydrogens of the LGN and O74 atoms of carbonyl groups in DDQ. The corresponding H49–O74 distances are 1.413 Å.

#### 2.5.3. Reduced Density Gradient (RDG) 

Johnson et al. [8] studied RDG, which describes the weak interactions that come from the quantum-mechanical electron density and its first derivatives in real space. The electron density value in a reduced density gradient, which is a fundamental dimensionless quantity called RDG, provides information about interaction strength and is defined as

(3) $$\mathrm{RDG}\left(r\right)=\frac{1}{2{\left(3\pi^{2}\right)}^{1/3}}\frac{\left|\nabla \rho \left(r\right)\right|}{\rho {\left(r\right)}^{4/3}},$$

where the ρ(r) is the electron density and ∇ρ(r) is the norm of the electron density vector. The sign of λ is used to distinguish bonded (λ_2_ < 0) from non-bonded (λ_2_ > 0). To better define the nature of the intermolecular interaction of LGN, the RDG analysis results plotted by the Multiwfn 3.4.1 package and VMD programs are shown in Figure 8 [44].

As shown in the scatterplot in Figure 8, many spikes are found in the region of −0.05–+0.05 a.u. As shown in Figure 8, negative values of sign (λ_2_)ρ (blue colour side of Figure 8) are indicative of attractive interactions with the other molecule, while positive values of sign (λ2)ρ (red colour side of Figure 8) indicate strong repulsion interactions (steric effect). 

As seen in Figure 8A, the isosurfaces of RDG among two molecules in the LGN-CHA complex show a blue ring between O(71)⋯H(49), which was suggested as a hydrogen bond, besides showing green-brown between O(71)⋯H(45), which indicates a strong Van der Waals interaction among two molecules in the complex.

While the LGN-DDQ complex was seen in Figure 8B, the isosurfaces of RDG between two molecules were observed to have a blue ring between H(49)⋯O(74), which was explained as a hydrogen bond, as well as two green-brown regions between H(50)⋯O(74) and H(50)⋯Cl(72), which were interpreted as two strong Van der Waals interactions among the two molecules in the complex. The significant steric effects can be seen in the red fusiform patches near the C=O bond and in the center of the phenyl rings, the steric effects that are the result of forces of repulsion between over-lapping electron clouds on aromatic rings. In addition to steric interactions, there are nonbonding interactions that affect the reactivity and shape of ions and molecules.

#### 2.5.4. Quantum Theory of Atoms in Molecules (QTAIM)

Bader’s proposed theory of atoms in molecules (AIM) has been extensively used to define the nature of diverse interactions in various molecular systems and to characterize the bonding interactions from the perspective of the real space functions as the electron density at the bond critical points (BCP) [45]. Topological parameters, including the Laplacian of electron density ∇2ρ(r), the electron density ρ(r), the Lagrangian kinetic energy G(r), the potential energy density V(r), the Hamiltonian kinetic energy (H(r) = G(r) + V(r)), and the bond energy E_int_ = V(r)/2, can be employed to investigate the parameters of hydrogen bonds that exist between compounds. 

In order to acquire the intramolecular BCPs and ring critical points (RCPs) between LGN–DDQ and LGN–CHA molecules for AIM studies, the optimised LGN–CHA and LGN–DDQ complexes were employed. The Multiwfn software was used for the AIM analysis calculation. Figure 8A–D provides an illustration of the molecular graph of the LGN–CHA and LGN–DDQ complexes in the RCP and BCP of the indicated molecular interactions produced by the Multiwfn programme. Table 2 provides a listing of the topological characteristics of these complexes in all of the BCPs and RCPs.

The hydrogen bond interactions, as described by Rozas et al. [46], can be divided into the following classifications:

(1) Weak bonds are specified by ∇2ρ(r) > 0 and H(r) >0
(2) Moderate bonds are specified by ∇2ρ(r) > 0 and H(r) < 0
(3) Strong bonds are specified by ∇2ρ(r) < 0 and H(r) < 0

The results of the electron density in Table 2 for the LGN–CHA complex showed that the interaction between LGN and CHA has two hydrogen bonds. The first bond is (H⋯O) BCP 149, which is classified as a moderate hydrogen bond by Rozas because the Laplacian of electron density ∇2ρ(r) is 0.145 a.u. (positive value), the Hamiltonian kinetic energy H(r) is −1.34 × 10^−4^ a.u. (negative value), and the ratio of |V(r)|/G(r) is greater than 1, indicating only moderate interactions between the atoms. 

Moreover, the value of the electron density (ρ(r)) is 0.038 a.u., which is extremely near to the values of the hydrogen bond in the standard suggested by Popelier and Bader [47].

The second bond of the LGN–CHA complex is BCP 121, which was a weak hydrogen bond due to the values of the Laplacian of electron density ∇^2^ρ(r) is 0.0181 a.u. (positive value) and the Hamiltonian kinetic energy H(r) is 6.84 × 10^−4^ a.u. (positive value). Furthermore, the value of the electron density (ρ(r)) is 0.0515 a.u. (close to the values of the standard hydrogen bond). 

The results for complex LGN-DDQ revealed that the interaction between LGN and DDQ was via three hydrogen bonds. The first bond is (H⋯O) BCP 126, which is a moderate hydrogen bond as the value of the Laplacian of electron density ∇2ρ(r) is 0.0213 a.u. (positive value), and the Hamiltonian kinetic energy H(r) is −2.60 × 10^−3^, indicating moderate interatomic interactions. Additionally, the value of the electron density (ρ(r)) is 0.047 a.u. (close to the values of the standard hydrogen bond). 

According to the value of the Laplacian of electron density ∇2ρ(r) between 0.0342 and 0.0183 a.u. (positive values) and the Hamiltonian kinetic energy H(r) between 1.14 × 10^−3^ and 1.04 × 10^−3^ (positive values), the second and third bonds of the LGN-DDQ complex (BCP 150 and BCP 163) were weak hydrogen bonds. Additionally, the values of electron density (ρ(r)) for these bonds are within the scope of 0.0995–0.0543 a.u. (close to the values of the standard hydrogen bond). As well, the value of ρ(r) at the H⋯O bond in LGN-CHA and LGN-DDQ complexes is shown to decrease as atomic separation increases in Table 2, which presents the topological properties of these complexes. 

In addition, the hydrogen bonds in both complexes were classified as non-covalent bonds due to the positive value of the Laplacian electron.

Further elongation of the non-covalent N−H bond of the electron acceptors in the complexes leads to a shorter H⋯O distance in the N−H⋯O fragment. The non-equivalence of the H⋯O bonds is also reflected in the energy values and topological parameters at the H⋯O critical bonding point. The H⋯O bonds shorter than 2 Å have negative H(r) values and can be classified as intermediate type interactions. 

In this study, three types of H-bonds were found for the different fragments and with different strengths. The first fragment in BCP is the NH electron acceptors (149 and 126 for LGN-CHA and LGN-DDQ, respectively). The H⋯O bonds are shorter than 2 and have a positive Laplacian of electron density ∇^2^ρ(r) and a negative H(r). These can be classified as non-covalent bonds (hydrogen bonds) and intermediate-type interactions.

The second C⋯H H-bond acceptor in BCP 121 (LGN-CHA) and 150 (LGN-DDQ) is the H⋯O bond, which has ∇2ρ(r) > 0, H(r) > 0, |V(r)|/G(r) < 1. From those results, they can be classified as closed-shell interactions (weak H-bonds of electrostatic character).

The third type of Cl⋯H−C was found in BCP 163 (LGN-DDQ), which was classified as a closed-shell interaction (weak H-bond of electrostatic character).

The RDG analysis was found to be in good agreement with the QTAIM results, as demonstrated by these results.

#### 2.5.5. Molecular Electrostatic Potential (MESP) Analysis

**Figure 9:**
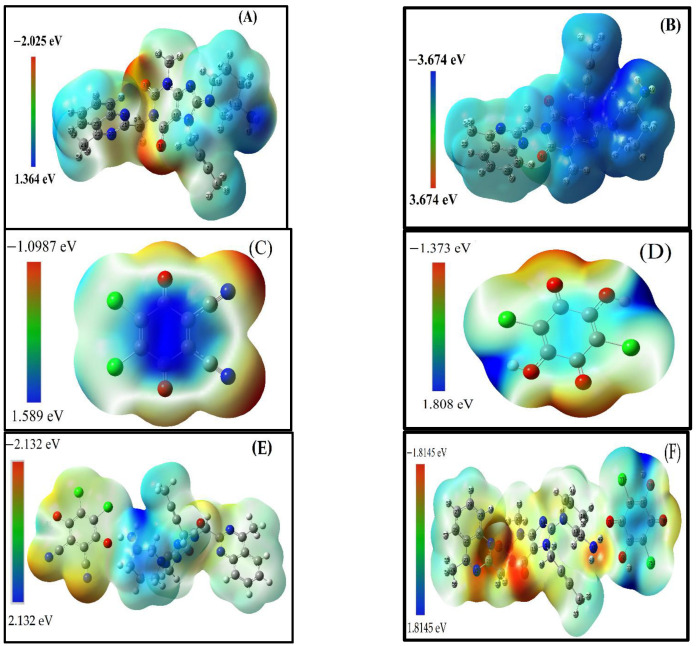
Molecular electrostatic potential (MESP) maps of (**A**) LGN, (**B**) LGN + 1, (**C**) DDQ, (**D**) CHA, (**E**) LGN⋯DDQ and (**F**) LGN⋯CHA.

Further analysis of their MESPs was performed in order to identify the different reaction sites on the surfaces of LGN, DDQ, and CHA. MEP maps use the local electron charge density to systematically investigate the ability of a molecule to interact. By referring to the mapped colours, the positions of strong electropositive and electronegative atoms were judged. These positions help to determine the active sites and determine the possible coordination modes of the template compound with the functional monomer. The MEP maps of LGN, DDQ and CHA are shown in Figure 9 with different colours. Red and yellow represent electron-rich regions, where the electrostatic potential is negative, and blue represents electron-deficient regions, where the electrostatic potential is positive [48,49].

As shown in Figure 9A, the red region (negative) of LGN is located mainly around O18, and O33, which is easily attributed to their lone pairs of electrons [49], indicating that O18 and O33 of LGN have higher energy levels. Hence, they are susceptible to attacks by electrophiles and can lose charge, making them electron donors. On the other hand, the blue region (positive) of LGN is mainly distributed around H48 and H49, revealing that the two atoms are at a lower energy level and can easily be attacked by nucleophiles, making them electron acceptors. The most common structure in the pH range from 2 to 10 has a positive charge. The protonation structures of LGN at different pH values (Figure S1 and Figure 9B), which were predicted using the Marvin sketch software, were used to match with the active site of the calculated structure via the MESP method.

In organic solvents such as MeCN, the most frequent structure is that shown Figure 9A, which has a neutral charge. However, in the charge transfer interaction of LGN, the active site when the acceptor gains charge from the donor was selected according to the theoretic calculation. These results of the distributed charge were matched with those in the predicted protonated structure.

MESP maps of this structure have a blue region (positive) found in the (π-hole) around C17, C16, C15, N34, N14, C6, N5, and N7, as well as σ-hole localized around the hydrogen atoms H48 and H49. 

The O atom in 1,4-benzoquinone derivatives as DDQ and CHA creates a negative region (A red region) of MESP, called the heap. In the DDQ in Figure 9C, the heaps (red region) found around Cl11, Cl12, O8 and O7 are the charge donors, while the blue region is found in the cloud above and below the molecular plane of the 1,4-benzoquinone, which are the electron acceptors or positive regions (π-hole). On the other hand, in the CHA in Figure 9D, the blue region found around H10 and H12 is the electron acceptor, and the red region is found around O8 and O7, which is the charge donor. The interaction of two complexes between the LGN with DDQ and with CHA were performed using the active area of MESP. The mechanism of formation of those complexes through the transfer of the charge from the heap region on the donor molecule to the positive region (π–hole) of 1,4-benzoquinone to produce the free radical negative ions on the acceptors beside the free radical positive ion on the donor follows that the O8 and O7 for the free radical negative ions (DDQ and CHA) interact as the hydrogen bond donors with the H48 or H49 of the free radical positive ion (LGN) as the hydrogen bond acceptors to give reddish brown complexes (Figure 9E,F).

In conclusion, the potential active sites of LGN are H48 or H49, while those of DDQ and CHA are O8 or O7, which are readily involved both electrophilically and nucleophilically. 

#### 2.5.6. UV–Visible Analysis

**Figure 10:**
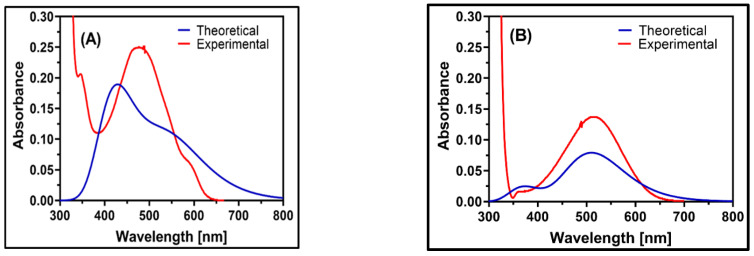
The experimental and theoretical UV spectra of: (**A**) LGN–DDQ (1:1) 1 × 10^–4^ M and (**B**) LGN–CHA (1:1) 1 × 10^–4^ M.

The UV–Vis spectra of the LGN–DDQ and LGN–CHA complexes were recorded in the wavelength range of 200–700 nm, as shown in Figure 10, and MeCN was used as a solvent for both theoretical and experimental analysis. The computational UV–visible absorption spectra have been calculated using the TD–DFT method based on the B3LYP/6-311G(d,p) level of theory. As well as the observed and computed wavelengths (λ), the corresponding electronic excitation energies, oscillator strength (ƒ), and the transition nature are calculated using the GaussSum 3 software listed in Table 3. The simulated UV absorption spectra of the LGN–DDQ and LGN–CHA complexes in MeCN solvent are displayed in Figure S3, which indicated the three transition states for the LGN–DDQ complex as 546.28, 424.48, and 371.66 nm, and for the LGN–CHA complex as 510.58, 420.17, and 371.73 nm.

According to Table 3, the LGN–CHA state has the most similar excitation energy for LGN complexes. The strong transition of the LGN–DDQ complex shows a maximum absorption wavelength experimentally at λ_max_ = 487.5 nm, while the excited state is equal to 424.48 nm with an oscillator strength of 0.0448. On the other hand, LGN–CHA complexes have an experimental maximum absorption wavelength of max = 514.0 nm, and their theoretical excited state wavelength is 510.58 nm, with an oscillator strength of *f* = 0.039. 

The strong transition of LGN–DDQ and LGN–CHA complexes shows a maximum absorption wavelength at λ_max_ = 487.5 nm and 514.0 nm (experimental), respectively, and 424.48 nm and 510.58 nm (theoretical), respectively, with an oscillator strength *f* = 0.0448 and 0.039, respectively. Considering calculated absorption spectra, the most extreme absorption wavelength relates to the electronic transition of the LGN–DDQ and LGN–CHA complexes from the highest occupied molecular orbital (HOMO) to the lowest unoccupied molecular orbital (LUMO) with 99% contribution for both complexes. The HOMO to LUMO 99.6% and 99.47% maximum contributions with bandgap energy of −65.242 and −70.589 kcal/mol show the charge transfer from the NH2 group in LGN to electronegative oxygen atoms in DDQ and CHA, respectively, as shown in Figure S2. The calculated absorption band of the LGN–DDQ and LGN–CHA complexes with the second highest intensity (λ_max_ = 510.58 nm and 424.47 nm, respectively) corresponds to the (HOMO → LUMO) and (HOMO–1 → LUMO) transitions with 99% contribution. The wavelengths with the lowest oscillator strength, 0.0000 and 0.0012, are assigned to the transition from the HOMO to LUMO + 1 and HOMO–1 to LUMO with 99.99 and 99.55 percent contributions.

### 2.6. Validation of Spectrophotometric Determination

#### 2.6.1. Linear Range and Sensitivity

In order to apply linear regression of the dataset, the least-squares methodology was used to build a calibration curve under optimal conditions of the spectrophotometric method [50]. It was found that the curve was linear for LGN-DDQ and LGN-CHA complexes with a high correlation coefficient in the range of 2.5–100 and 5–100 µM, respectively. Table 4 depicts the linear fitting parameters (intercept, slope, and correlation coefficient) for a given linear fit. Following the guidelines of the International Conference on Harmonization (ICH), the limit of detection (LOD) and limit of quantitation (LOQ) were calculated [51]. It was found that for LGN-DDQ and LGN-CHA complexes the LOD values were 1.0844 and 1.4406 while and LOQ values were 3.2861 and 4.3655 µM, respectively. Table 4 contains a summary of the calibration and validation parameters for the developed spectrophotometric method.

#### 2.6.2. Precision and Accuracy

Using samples of LGN solution at varying concentration levels, the precisions of the recommended spectrophotometric method were assessed, and the results are summarized in Table 5. For intra– and inter–assay precision, the relative standard deviations (RSD) for LGN–DDQ were 0.374–1.235% and 0.383–1.177%, respectively. While for LGN–CHA were 0.411–1.355% and 0.681–1.107%, respectively. The assay’s good precision was confirmed by these low RSD values. Recovery studies at the same LGN-DDQ and LGN–CHA complexes concentration levels utilized in the precision studies were conducted to assess the assay accuracy. The recovery values ranged from 98.798 to 101.118 percent and 98.718 and 102.725 (Table 5), demonstrating that the proposed assay is highly accurate.

## 3. Materials and Methods

### 3.1. Reference Samples and Reagents

The authentic reference standard of LGN (>99.00% *w*/*w*) was provided by Boehringer Ingelheim pharmaceutical company (Germany). All of the reagents, including solvents, used in the investigation were of an analytical grade (Fisher Scientific, California, CA, USA).

### 3.2. Apparatus

UV–Vis spectrophotometer (UV-1601 PC, made by Shimadzu in Kyoto, Japan), double beam, with matching 1 cm quartz cells.

### 3.3. LGN Standard Preparation

The stock standard LGN solution was made by dissolving an accurately weighed quantity (23.63 mg) of the standard material in 10 mL of MeCN. The final concentration of the solution was 5 mM. Then, MeCN was added to dilute the solution until it had the right amount of LGN for each of the next tests.

### 3.4. The Molar Ratio and the Association Constant

In order to determine the association constant of the CTC, the Benesi–Hildebrand technique [42] was used to select DDQ or CHA solutions ranging from 1 to 4 μM and a constant concentration of LGN of 3 μM. The molar ratios of LGN: DDQ and LGN:CHA in the reaction were determined using Job’s continuous variation approach [41]. Equimolar solutions (1 μM) of LGN, DDQ, and CHA reagent were utilized.

### 3.5. Computational

#### 3.5.1. Calculations of Electronic Structure

All molecular orbital theory and density functional theory calculations were carried out with the Gaussian 09 software package as previously reported [52,53]. Geometries and frequencies of all species were calculated using the B3LYP functional. All species were optimized in vacuo using the B3LYP/6-311G(d,p) method [54,55]. All molecular structures were characterized with a single imaginary frequency and a minimum of zero imaginary frequencies. 

Harmonic frequency calculations have been performed at all the mentioned levels of theory to characterize the nature of the stationary point. All these structures were found to be local minima with all real values of the Hessian matrix. 

#### 3.5.2. Interaction Energies (IE)

The binding energies were calculated with correction for the basis set superposition error (BSSE) with the ‘‘Counterpoise = N” option using the Boys–Bernardi counterpoise technique [56]. The optimization of the molecular geometries has been performed by imposing the highest symmetry point group. The interaction energies (IE) of these molecules were calculated by the energy difference between the whole molecule and the sum of the energies of separated fragments using Equation (4)

(4) $$\mathrm{IE}=E_{\mathrm{whole}-\mathrm{molecule}}-E_{\mathrm{LGN}}-E_{X},$$

where the whole-molecule refers to the LGN-X system and X represents the DDQ or CHA molecules shown in Schemes S1 and S2. All local minimum structures were taken, and zero-point and BSSE corrections were included in this calculation.

#### 3.5.3. Non-Covalent Interaction (NCI) Index

The non-covalent interaction (NCI) index, recently introduced by Yang et al. [57], provides qualitative visualization of noncovalent interaction by mapping the non-covalent interaction zone in real space qualitatively. The method is based on two scalar fields, i.e., electron density (q) and reduced density gradient (RDG, s), to map the bonding properties.

The combination of s and q provides a rough partitioning of real space into bonding regions: low-s–low-q to non-covalent interactions and low-s–high-q to covalent interactions. The NCI plot is very helpful in distinguishing H-bonding, van der Waals, and steric interactions. At low-q and low-s, the non-covalent interaction area is defined. The low-s value at the negative low-q is an attractive zone. The low-s value with a positive low-q is a repulsive zone. We have carried out NCI analysis using the Multiwfn program [58].

#### 3.5.4. Quantum Theory of Atoms in Molecules (QTAIM)

The topology of electron density in a molecule can be analysed using Bader’s quantum theory of atoms in molecules (QTAIM) [45,59,60,61]. Generally, for covalent interactions (also referred to as “open-shell” or “sharing” interactions), the electron density at the bond critical point (BCP), ρb, is large (>0.2 a.u) while its Laplacian, ∇^2^ρ, is large and negative. On the other hand, for closed-shell interactions (e.g., ionic, van der Waals, or hydrogen bonds), ρb is small (<0.1 a.u.) and ∇^2^ρ is positive. QTAIM is one of the appropriate approaches to analyse different intra- and intermolecular interactions since their properties are expressed as characteristics of a real electron density of a system analysed. We have carried out QTAIM analysis at the B3LYP/6-311G(d,p) level of theory using the Multiwfn [58] program.

#### 3.5.5. Molecular Electrostatic Potential (MESP)

Molecular electrostatic potential (MESP) is a well-established tool for predicting the reactive behaviour of various chemical systems [62,63,64,65]. Tomasi et al. pioneered the application of MESP for understanding intermolecular interaction [66,67]. In the realm of MESP, the most negative value of the MESP (V_min_) is characterized as the lone pair or π electron density or “heap”, while the most positive region (V_max_) is characterized as the electron deficient region or “hole” [68,69]. The interaction of this hole with the heap is responsible for the noncovalent interaction present in these systems. Gadre and co-workers have utilized the MESP tool to predict the structures and interaction energies of some lone pair-π interactions in substituted benzene. We have also applied these MESP tools to systematically characterize the position of hole and heap in LGN, DDQ, CHA and their complexes to understand the structure of these noncovalent complexes. MESP calculations were performed using the Multiwfn program [59,70]. The protonation structures of LGN at various pH values were predicted using Marvin sketch software [71].

#### 3.5.6. Ultraviolet–Visible Spectra (UV–Vis)

UV–Vis spectra were computed by solving time-dependent density functional theory (TD-DFT) [72,73] equations and evaluated using the GausSum 3 program [74]. Chemical models were used to generate theoretical UV–Vis spectra in the presence of MeCN as a solvent using the conductor-like polarizable continuum model (CPCM) approach and the Gaussian 09 program [75].

## 4. Conclusions

The color complexes of LGN with DDQ and CHA were synthesized and characterized with visible spectral and compared to theoretical support with the support of Gaussian software. A UV–visible study showed that LGN-DDQ and LGN-CHA complexes form when they react in MeCN. This was shown by the appearance of a new unique absorption band at 487 nm and 514 nm. The molecular stoichiometry of the complex was determined to be 1:1. The λ_max_ and ε of the complex were dependent on both the polarity index and the dielectric constant of the solvent utilized for the reaction. The molecular electrostatic potential study of the LGN reveals that it is the most susceptible to a nucleophile attack. In the visible spectrum, the theoretical and experimental peaks are almost the same. We observed that there are interactions between LGN molecules and DDQ and CHA molecules using atoms in molecule (AIM) and reduced density gradient (RDG) studies. These interactions include hydrogen bond interactions, van der Waals contacts, and spatial effects, which are evidence of the contribution of non-covalent interactions in the formation of complexes. The method reported in this work is regarded as the standard spectrophotometric method for measuring LGN. The assay is distinguished by its high throughput, which makes it possible to conduct analysis on a large quantity of samples.

## Figures and Tables

**Table 1:** Interaction energies without and with the BSSE ((∆E)_raw_, and (∆E)_corrected_ and ∆EBSSE, respectively, in kcal mol^−1^ at the B3LYP/6-311G(d,p) level of theory.

Complexes	(∆E) Raw kcal/mol	∆EBSSE	(∆E) Corrected kcal/mol
LGN-DDQ	–75.32	0.00338	–73.20
LGN-CHA	–77.30	0.00638	–73.30

**Table 2:** Calculated Hydrogen Bonding Interaction Parameters: Hydrogen Bond Distance ((r, Å), Electron Density, Electron Density Laplacian and Energy Density at the H⋯O BCPs (ρ(r), ∇^2^ρ(r), and H(r), au), |V(r)|/G(r) Ratio.

Complex	BCP	Bond and Bond Distance (Å)	ρ(r) (a.u.)	K(r) (a.u.)	V(r) (a.u.)	H(r) (a.u.)	∇^2^ρ(r) (a.u.)	|V(r)|/G(r) (a.u.)
LGN–CHA	121	O(71)⋯H(45)-C(10) 2.78298	5.15 × 10^−3^	−6.84 × 10^−4^	−3.16 × 10^−3^	6.84 × 10^−4^	1.81 × 10^−2^	0.823
	149	O(71)⋯H(49)-N(12) 1.76989	3.87 × 10^−2^	1.34 × 10^−4^	−36.6 × 10^−3^	−1.34 × 10^−4^	14.5 × 10^−2^	1.003
LGN–DDQ	126	N(12)-H(49)⋯O(74) 1.67704	4.70 × 10^−2^	2.60 × 10^−3^	−4.73 × 10^−2^	−2.60 × 10^−3^	2.13 × 10^−2^	1.057
	150	C(13)-H(50)⋯O(74) 2.46133	9.95 × 10^−3^	−1.14 × 10^−3^	−6.27 × 10^−3^	1.14 × 10^−3^	3.42 × 10^−2^	0.847
	163	C(13)-H(50)⋯Cl(72) 2.97070	5.43 × 10^−3^	−1.04 × 10^−3^	−2.49 × 10^−3^	1.04 × 10^−3^	1.83 × 10^−2^	0.705

**Table 3:** UV–Vis band gap energy E (eV) and oscillator strength (*f*) for the LGN-DDQ and LGN-CHA molecule calculated at TD-DFT/B3LYP method using MeCN solvent.

Complex	Experimental		Theoretical (TD–DFT Calculation)			
	(λ_max_) nm	Band Gap (eV)	Excited State	Band Gap (eV)	Oscillator Strength (*f*)	* Transition State (Coefficient)
LGN⋯DDQ			546.28	2.27	0.0245	HOMO → LUMO (99%)
	487.5	2.54	424.48	2.92	0.0448	HOMO → LUMO + 1 (100%)
	346.0	3.58	371.66	3.34	0.0000	HOMO − 1 → LUMO (99%)
LGN⋯CHA.	514.0	2.41	510.58	2.43	0.0039	HOMO → LUMO (100%)
	487.5	2.54	420.17	2.95	0.0000	HOMO − 1 → LUMO (99%)
	379.0	3.27	371.73	3.34	0.0012	HOMO → LUMP + 1 (100%)

* Transition state is significant contributions for electronic transitions from the H-highest occupied MO (HOMO) to the L-lowest unoccupied MO (LUMO).

**Table 4:** Calibration and validation parameters for the determination of LGN by the proposed spectrophotometric method based on its formation of coloured complexes with DDQ and CHA.

Parameter	LGN-DDQ	LGN-CHA
Linear range (µM)	2.5–100	5–100
Intercept	0.04729	0.01416
Standard deviation of intercept	0.0009602	0.0005387
Slope	0.002922	0.001234
Standard deviation of slope	1.980 × 10^–5^	1.048 × 10^–5^
Correlation coefficient	0.9997	0.9996
LOD (µM)	1.0844	1.4406
LOQ (µM)	3.2861	4.3655

**Table 5:** Precision and accuracy of the proposed spectrophotometric method at different LGN with DDQ and CHA complexes concentration levels.

Taken Concentration (µM)	Precision: Relative Standard Deviation (%)		Accuracy: Recovery (% ± SD)
	Intra–Assay	Inter–Assay	
**LGN–DDQ complex**			
10	1.039	0.383	101.12 ± 0.41
30	0.733	0.839	98.91 ± 0.62
50	0.374	1.017	98.80 ± 0.90
100	1.235	1.177	99.57 ± 1.25
**LGN–CHA complex**			
10	0.411	0.681	99.34 ± 0.29
30	1.355	1.107	98.72 ± 0.86
50	1.013	0.852	102.73 ± 1.72
100	0.710	0.751	100.756 ± 1.47

## Data Availability

Not applicable.

## Supplementary Materials

The following supporting information can be downloaded at: https://www.mdpi.com/article/10.3390/molecules27196320/s1, Figure S1: Different hypothetical protonation structures of LGN expected in various pH values. Where panel (A) represents the molecular structures of the various proton states of LGN and panel (B) represents the distribution of LGN microspecies by pH value. There is a match between curves’ colours and their corresponding chemical structures; Figure S2: (A&B) The frontier molecular orbitals and related energies (in gas phase); Figure S3: The theoretical UV-Vis spectra of (A) LGN–DDQ complex and (B) LGN–CHA complex molecule gas phase, in acetonitrile; Scheme S1: Formation of LGN and DDQ complex from the free radical ions coupling in ACN at room temperature; Scheme S2: Formation of LGN and CHA complex from the free radical ions coupling in ACN at room temperature.
