# Supplementary material for: Thermodynamic and Computational (DFT) Study of Non-Covalent Interaction Mechanisms of Charge Transfer Complex of Linagliptin with 2,3-Dichloro-5,6-dicyano-1,4-benzoquinone (DDQ) and Chloranilic acid (CHA)
Source: Molecules. 2022 Sep 25;27(19):6320. doi: 10.3390/molecules27196320 (PMC9572772; doi:10.3390/molecules27196320)
Supplement: Supplementary file 1 [file molecules-27-06320-s001.zip › molecules-1922241-supplementary.pdf]

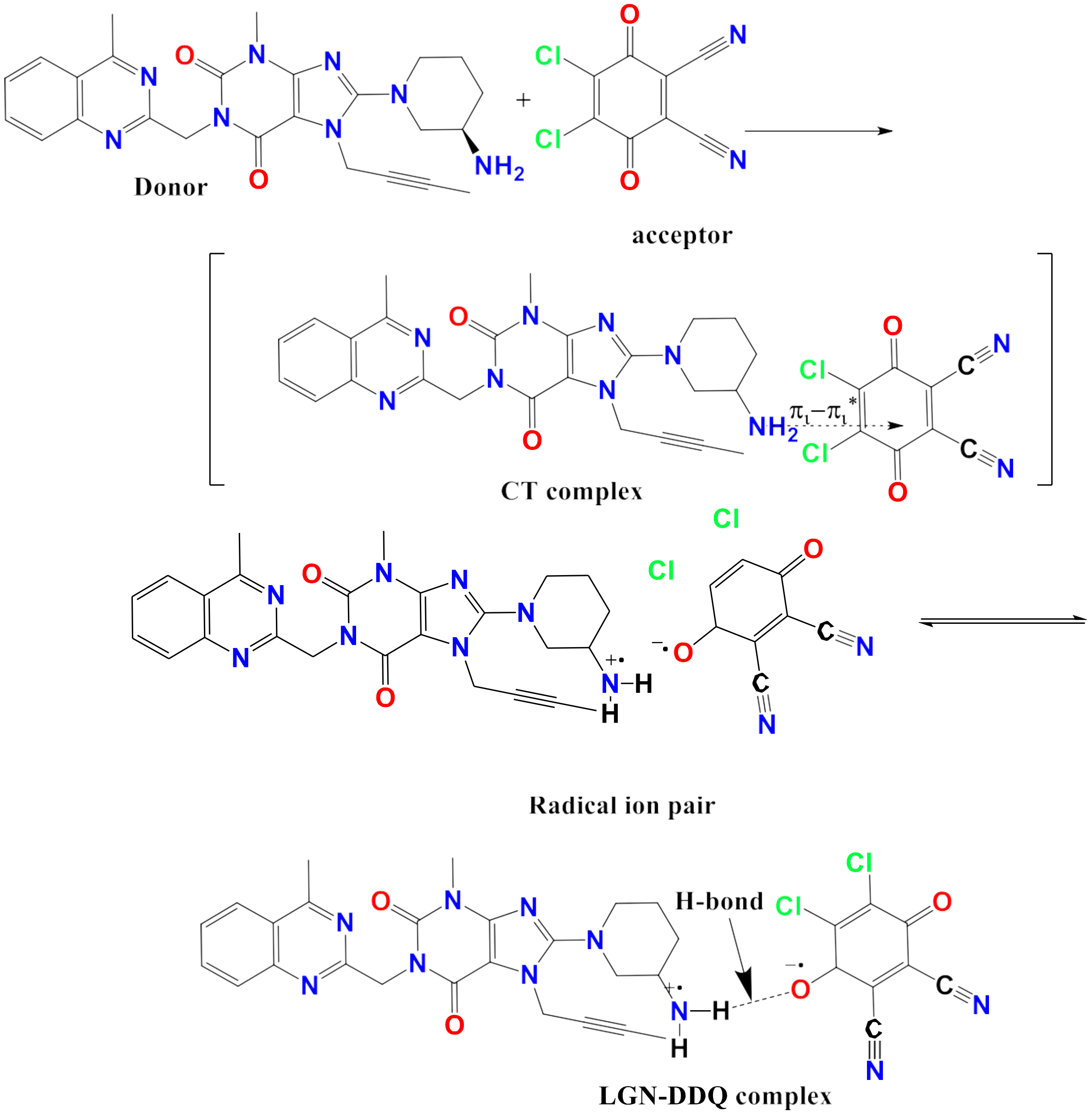

Scheme S1: Formation of LGN and DDQ complex from the free radical ions coupling in ACN atroom temperature.

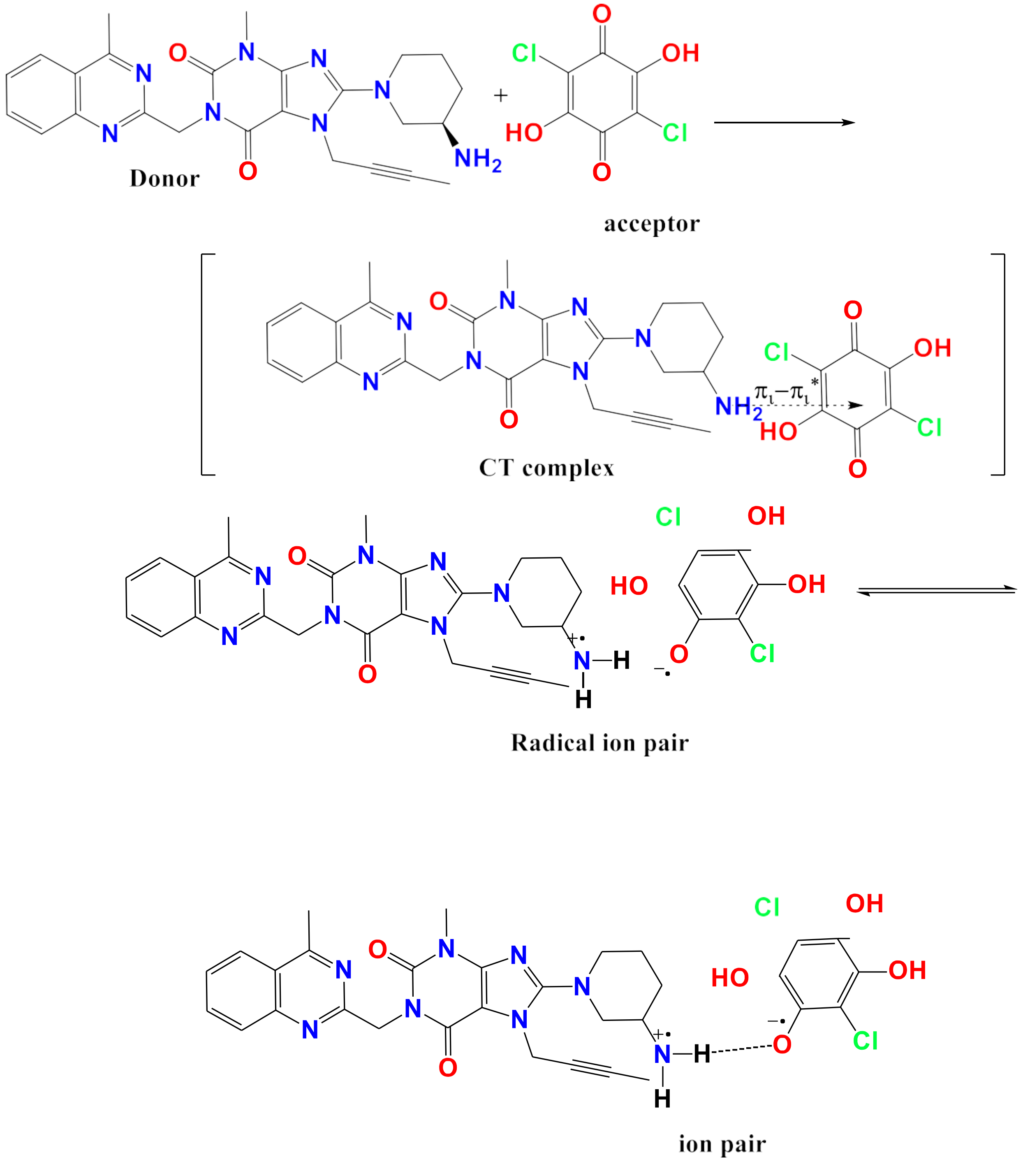

Scheme S2: Formation of LGN and CHA complex from the free radical ions coupling in ACN atroom temperature.

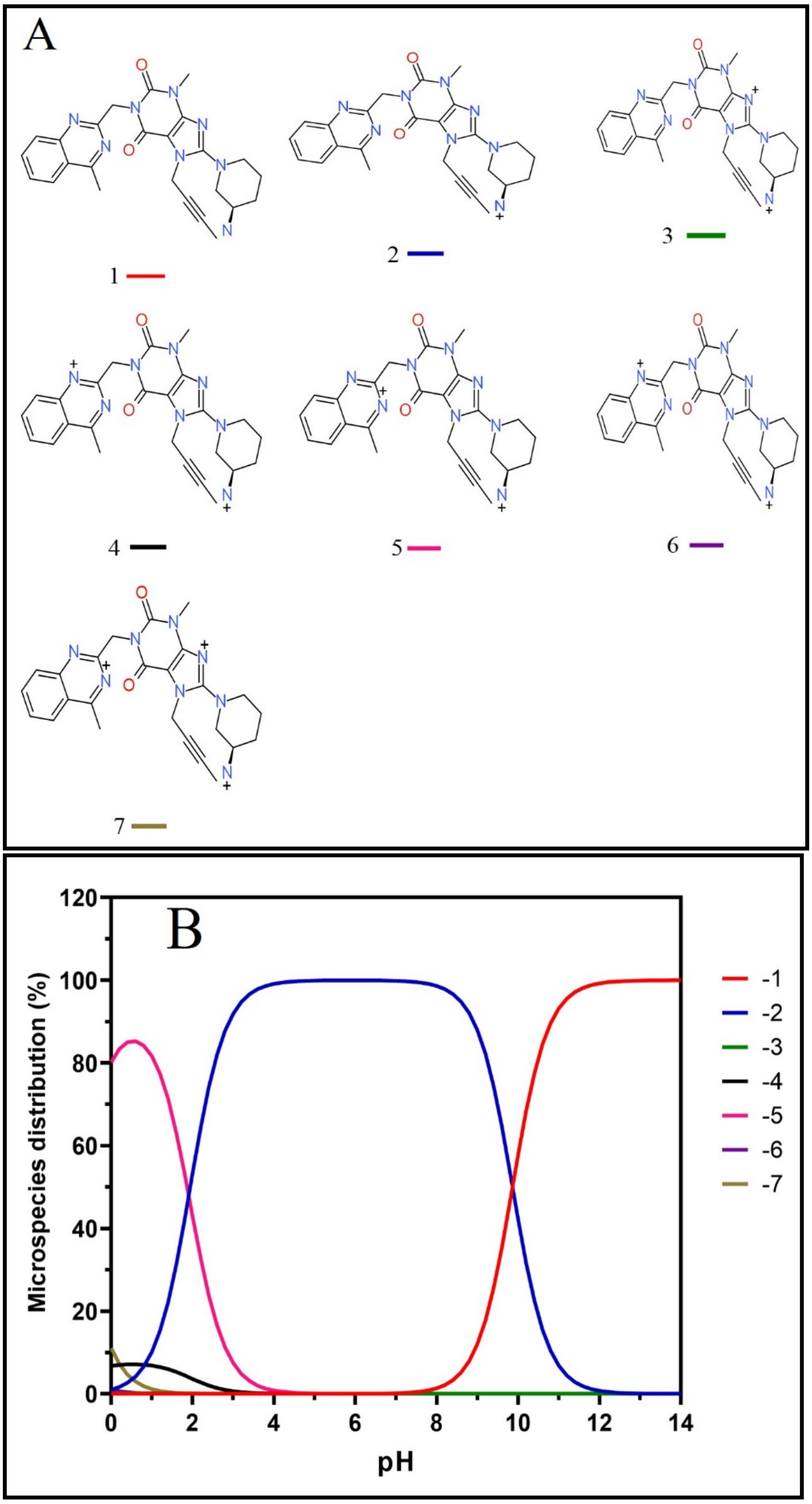

Figure S1. Different hypothetical protonation structures of LGN expected in various pH values. Where panel (A) represents the molecular structures of the various proton states of LGN and panel (B) represents the distribution of LGN microspecies by pH value. There isa match between curves' colours and their corresponding chemical structures.

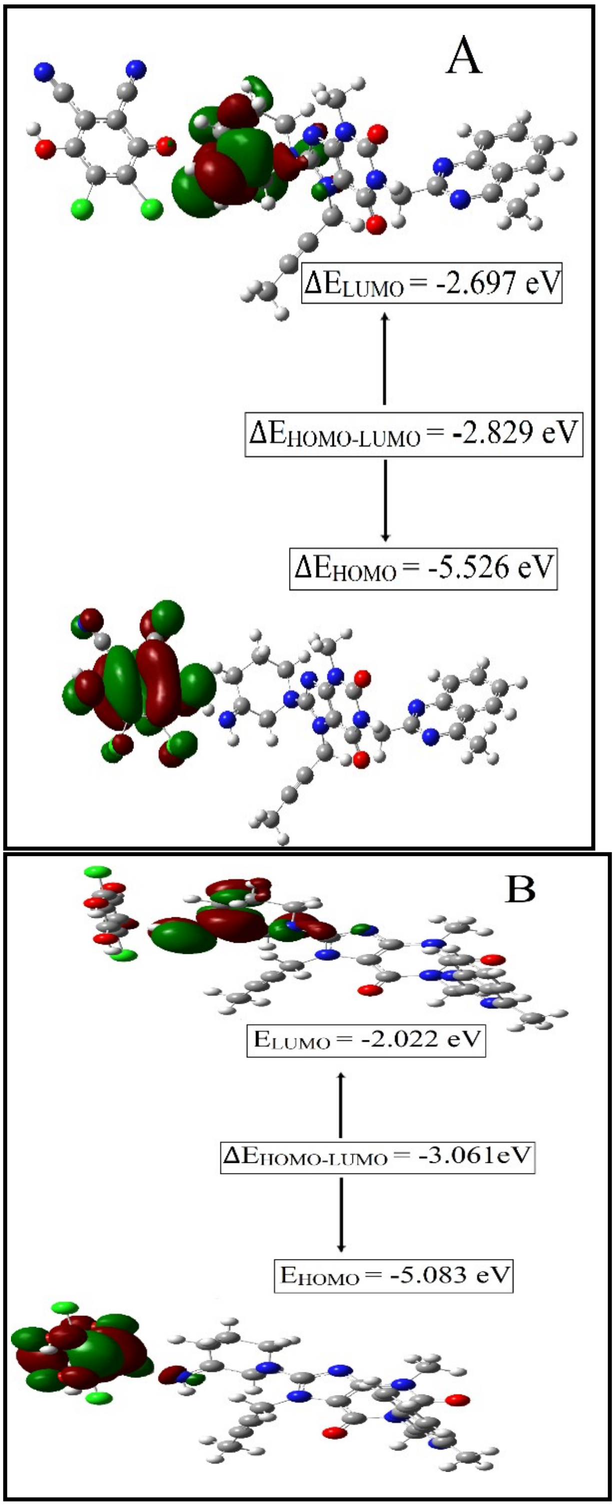

Figure S2. The frontier molecular orbitals and related energies (in gas phase).

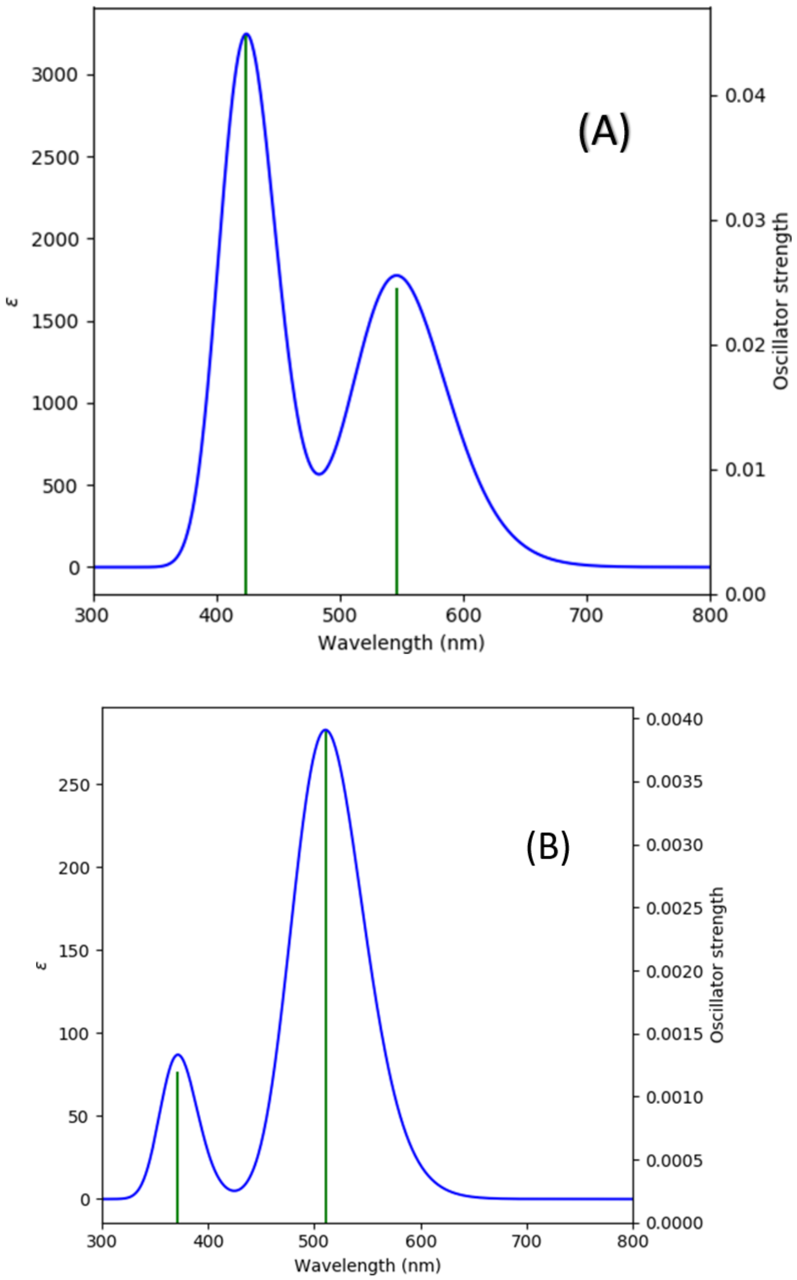

Figure S3. The theoretical UV-Vis spectra of (A) LGN–DDQ complex and (B) LGN–CHAcom-plex molecule gas phase, in acetonitrile.
